# Supplementary material for: Niclosamide–Clay Intercalate Coated with Nonionic Polymer for Enhanced Bioavailability toward COVID-19 Treatment
Source: Polymers (Basel). 2021 Mar 26;13(7):1044. doi: 10.3390/polym13071044 (PMC8036780; doi:10.3390/polym13071044)
Supplement: Supplementary file 1 [file polymers-13-01044-s001.pdf]

polymers

MDPI

Supplementary Material

Niclosamide–Clay Intercalate Coated with Nonionic Polymer for Enhanced Bioavailability toward COVID-19 Treatment

Seungjin Yu [1,2,†], Huiyan Piao [2,†], N. Sanoj Rejinold [2], Geunwoo Jin [3], Goeun Choi [2,4,5,*] and Jin-Ho Choy [2,6,7,*]

1 Department of Chemistry, College of Science and Technology, Dankook University, Cheonan 31116, Korea; 32182808@dankook.ac.kr
2 Intelligent Nanohybrid Materials Laboratory (INML), Institute of Tissue Regeneration Engineering (ITREN), Dankook University, Cheonan 31116, Korea; 12192032@dankook.ac.kr (H.P.), sanojrejinold@dankook.ac.kr (N.S.R.)
3 R&D Center, CnPharm Co., LTD., Seoul 03759, Korea; geunwoo.jin@cnpharm.co.kr
4 College of Science and Technology, Dankook University, Cheonan 31116, Korea
5 Department of Nanobiomedical Science and BK21 PLUS NBM Global Research Center for Regenerative Medicine, Dankook University, Cheonan 31116, Korea
6 Department of Pre-medical Course, College of Medicine, Dankook University, Cheonan 31116, Korea
7 Tokyo Tech World Research Hub Initiative (WRHI), Institute of Innovative Research, Tokyo Institute of Technology, Yokohama 226-8503, Japan
* Correspondence: goeun.choi@dankook.ac.kr (G.C.); jhchoy@dankook.ac.kr (J.-H.C.)
† These authors contributed equally to this work.

Table S1. Rate constants and r^2 coefficients obtained from fitting analyses based on several kinetic equations.

Kinetic Equation	NIC(1.0)-MMT	NIC(1.0)-MMT-E	NIC(1.3)-MMT-E
	**First order : $\ln(C_t/C_0) = -k_d t$**		
k_d	0.02928	0.2889	0.2337
r^2	0.9310	0.9806	0.3789
	**Parabolic diffusion : $(1 - C_t/C_0)/t = k_d t^{-0.5} + a$**		
k_d	1.0633	0.4478	1.8991
a	-0.2984	-0.0349	-0.6347
r^2	0.9531	0.9896	0.9485
	**Freundlich : $\log(1 - C_t/C_0) = \log(k_d) + a\log t$**		
k_d	0.8113	0.6670	1.0478
a	0.2087	0.4305	0.0483
r^2	0.9883	0.9950	0.6638
	**Elovich : $1 - C_t/C_0 = a\ln t + b$**		
k_d	0.1368	0.1988	0.0420
a	0.6393	0.4587	0.9010
r^2	0.9839	0.9676	0.6789

† These authors contributed equally to this work.
C_t = the amount of guest in the NIC at t min; C_0 = the amount of guest in the NIC at 0 min; k_d = the rate constant of release, a, b, n = constant.

In order to understand the release mechanism of NIC from the NIC loaded MMT hybrids, the observed release profiles were fitted to four kinetic models as plotted in **Figure S1** and the obtained rate constants (k_d) and r^2 values were summarized in **Table S1**. [37,39] Considering the r^2 value, the release curves of NIC from NIC(1.0)-MMT-E nanohybrids were well fitted with all the four kinetic models, i.e., First-order (0.9806), Parabolic diffusion (0.9896), modified Freundlich (0.9950), and Elovich model (0.9676) (**Table S1**). However, the results showed that the release profiles were best fitted to Parabolic diffusion and Freundlich models. Moreover, from the parabolic diffusion model, the release rate coefficients for NIC(1.0)-MMT and NIC(1.3)-MMT-E hybrids were determined to be 0.9531 and 0.9485, respectively, which were less than NIC(1.0)-MMT-E

hybrid (0.9896). The calculation with modified Freundlich model revealed that the release coefficients for NIC(1.0)-MMT (0.9883) and NIC(1.3)-MMT-E (0.6638) hybrids were calculated to be less than that for NIC(1.0)-MMT-E (0.9950) hybrid. All the calculation results suggested strongly that the release of the intercalated NIC from NIC(1.0)-MMT-E hybrid was a kind of diffusion-controlled processes or heterogeneous diffusion processes.

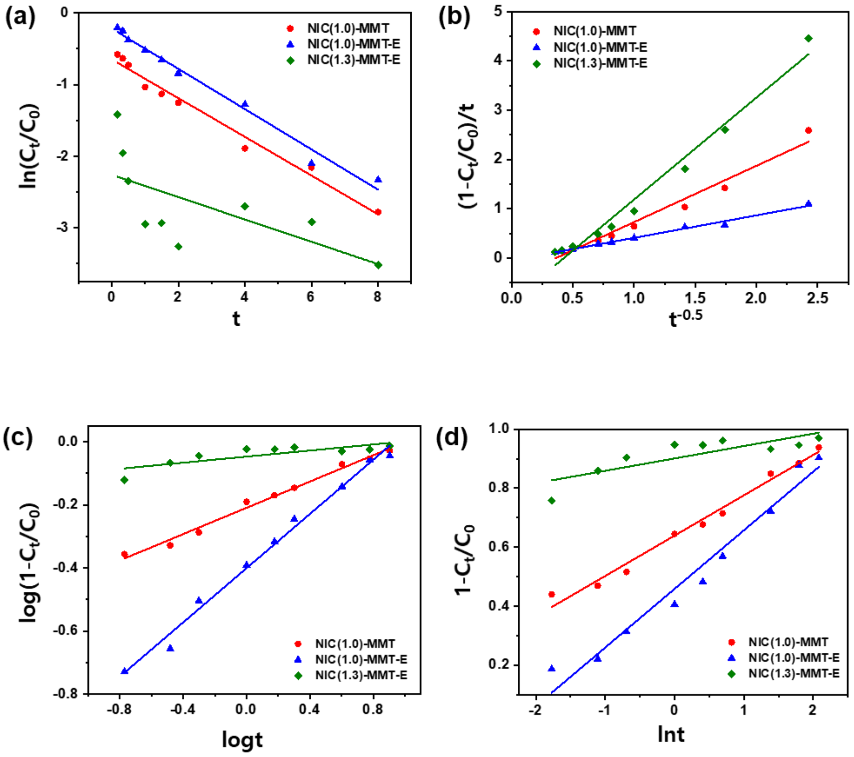

Figure S1. Plots of kinetic equation of (**a**) First-order kinetic model, (**b**) Parabolic diffusion model, (**c**) modified Freundlich model, and (**d**) Elovich model for the release of NIC from NIC(1.0)-MMT (red), NIC(1.0)-MMT-E (blue) and NIC(1.3)-MMT-E (green) hybrids.
